# Microbiota and Its Role on Viral Evasion: Is It With Us or Against Us?

**DOI:** 10.3389/fcimb.2019.00256

**Published:** 2019-07-18

**Authors:** Carolina Domínguez-Díaz, Alejandra García-Orozco, Annie Riera-Leal, Jorge Ricardo Padilla-Arellano, Mary Fafutis-Morris

**Affiliations:** ^1^Doctorado en Ciencias Biomédicas, Con Orientaciones en Inmunología y Neurociencias, Universidad de Guadalajara, Guadalajara, Mexico; ^2^Centro de Investgación en Inmunología y Dermatología (CIINDE), Zapopan, Mexico; ^3^Centro Universitario de Ciencias de la Salud, Universidad de Guadalajara, Guadalajara, Mexico

**Keywords:** microbiota, microbioma, viral evasion, microbiota-virome interaction, microbiota and antiviral immune defense

## Abstract

Viruses are obligate intracellular pathogens that require the protein synthesis machinery of the host cells to replicate. These microorganisms have evolved mechanisms to avoid detection from the host immune innate and adaptive response, which are known as viral evasion mechanisms. Viruses enter the host through skin and mucosal surfaces that happen to be colonized by communities of thousands of microorganisms collectively known as the commensal microbiota, where bacteria have a role in the modulation of the immune system and maintaining homeostasis. These bacteria are necessary for the development of the immune system and to prevent the adhesion and colonization of bacterial pathogens and parasites. However, the interactions between the commensal microbiota and viruses are not clear. The microbiota could confer protection against viral infection by priming the immune response to avoid infection, with some bacterial species being required to increase the antiviral response. On the other hand, it could also help to promote viral evasion of certain viruses by direct and indirect mechanisms, with the presence of the microbiota increasing infection and viruses using LPS and surface polysaccharides from bacteria to trigger immunosuppressive pathways. In this work, we reviewed the interaction between the microbiota and viruses to prevent their entry into host cells or to help them to evade the host antiviral immunity. This review is focused on the influence of the commensal microbiota in the viruses' success or failure of the host cells infection.

## Introduction

The mucosal surfaces of the human body contain complex communities of microorganisms collectively referred to as microbiota; these bacteria are a key factor in health and disease due to their participation in the development of the immune system and their host-protection against pathogens (Human Microbiome Project Consortium, 2012a,b; Lloyd-Price et al., 2016).

Viruses are a large and heterogeneous group of dependent biological agents that require the host-cell machinery to replicate. Most viruses are identified based on their capacity and mechanisms used to produce disease; however, healthy individuals harbor viral communities that do not cause directly known pathologies. These viral communities are known as the human virome (Rohwer et al., 2009). The coexistence of viruses and bacteria within the microbiome encourages the study of viral evasion mechanisms that provide immune system tolerance to these pathogens. These mechanisms are undoubtedly also used during the pathophysiology of viral diseases (Abeles and Pride, 2014).

## Microbiota Against Viral Infection

Since the discovery that gut bacteria instruct host immunity, i.e., they restrict pathogen proliferation, it would seem logical to think that the intestinal microbiota would also play a predominant role in viral etiology infection inhibition. Studies reveal that commensal bacteria are crucial in maintaining immune homeostasis and immune responses at mucosal surfaces (Ichinohe et al., 2011). Mucous membranes are the gateway to many pathogens, including viruses. For example, intestinal microorganisms promote maturation of the secondary lymphoid organs within the gastrointestinal tract, which is the first line of defense of the intestinal mucosa (Karst, 2016). Germ-free mice are unable to mount an efficient immune response against pathogens due to immature intestinal lymphoid structures (Hooper et al., 2012; Kamada and Núñez, 2014).

Given the complexity of the microenvironment in mucosal surfaces, it makes sense that the most studied bacteria and virus interactions are the ones involving the intestinal microbiome. The protective role of commensal bacteria, mainly probiotics, is well-established; however, in its interactions with viruses, more studies are needed. The *Lactobacillus* genus can inhibit murine norovirus (MNV) replication *in vitro*, which could be mediated by the increased expression of IFNβ and IFNγ. *In vivo* models show that these bacteria are decreased during MNV infection, though with the aid of retinoic acid treatment, it is possible to avoid this effect. It has been hypothesized that the antiviral effects of vitamin A (and consequently, retinoic acid) are mediated by the *Lactobacillus* genus due to interferon production (Lee and Ko, 2016).

Bacterial flagellin is efficient against rotavirus (RV) infection because it activates Pattern Recognition Receptors (PRR), TLR5 and NLRC-4, that stimulate the release of interleukin-22 (IL-22) and IL-18; the former induces normal epithelium proliferation, while the latter induces infected epithelial cell apoptosis (Zhang et al., 2014). *Bifidobacterium breve* and a mixture of this probiotic with galactooligosaccharides and fructooligosaccharides have a preventive effect against RV infection by increasing the production of IFNγ, IL-4, TNFα, and TLR2 expression, while also decreasing the tolerogenic response (Rigo-Adrover et al., 2018), thereby enhancing the mucosal defense against this pathogen.

Immunoregulation and reinforcement of the intestinal barrier through the relationship between commensal bacteria, probiotics, epithelial, and immune cells, are established physiological processes mediating the antiviral effects of the microbiota. The enteric microbiota regulates increased mucus production and synthesis of potential antiviral compounds, likereactive oxygen species and defensins, that inhibit local viral replication (Monedero et al., 2018).

Gut microbiota could also have distal protective effects on antiviral responses. There is evidence of the role of inflammasome activation in the immune defense against influenza virus infection (Allen et al., 2009; Ichinohe et al., 2009); it induces dendritic cell migration to the local lymph node to stimulate an influenza-specific T-cell response in the lung (Ichinohe et al., 2011; Wilks and Golovkina, 2012). The commensal gut microbiota regulates the respiratory mucosa immunity against respiratory influenza virus through the IgA secretion, and the proper activation of inflammasomes, Th1 cells, and CTLs, and through the upregulation of TLR7 signaling in the respiratory mucosa (Ichinohe et al., 2011; Wu et al., 2013). Steed et al. (2017) demonstrated that desaminotyrosine, a microbial metabolite, enhances type I interferon (IFN-I) signaling and protects against influenza pathogenesis.

Gut probiotics like *Lactobacillus paracasei* and *Lactobacillus plantarum* increase pro-inflammatory cytokines like IL-33, IL-1α, IL-β, IL-12, and IFNγ during influenza virus infection. There is also an increase in the presence of innate immune cells in the lungs such as NKs, macrophages, and dendritic cells. These probiotics were also able to diminish the inflammatory response in the lungs by an IL-10 increase, thereby controlling the antiviral response (Park et al., 2013; Belkacem et al., 2017). The crosstalk between the gut and airway bacteria through the gut-lung axis could explain how the intestinal bacteria are able to improve antiviral immunity since gut microbial metabolites could stimulate immune cells that can move to distal locations to mediate the antiviral response.

On the respiratory surface, airway bacteria protect against viral infections. *Staphylococcus aureus* stimulates the recruitment of peripheral CCR2^+^ CD11b^+^ monocytes and their subsequent maturation into M2 macrophages, through the activation of TLR2 signaling during influenza infection. This mechanism dampens influenza-mediated acute lung injury (Wang et al., 2013). The respiratory commensal bacteria, *Corynebacterium pseudodiphtheriticum* modulates the TLR3 antiviral response against Respiratory Syncytial Virus (RSV), enhancing the production of TNFα, IL-6, IFNγ, and IFNβ through the increase of T-cell subpopulations that produce these cytokines (Kanmani et al., 2017).

The vaginal mucosa is dominated by bacteria from the *Lactobacillus* genus. Vaginal microbial communities dominated by *Lactobacillus crispatus* were associated with a decreased HIV infection in South African women (Gosmann et al., 2017). *L. crispatus, Lactobacillus gasseri*, and *Lactobacillus vaginalis* inhibit HIV-1 replication in *ex vivo* cervico-vaginal tissue culture. These effects are mediated through acidification of the medium and lactic acid production, as well as their binding to the virus in order to reduce the free virions in the tissue (Ñahui Palomino et al., 2017). Lactic acid and acidic pH increase the production of anti-inflammatory cytokines, preventing the production of pro-inflammatory cytokines by epithelial cells and, with this, the inflammation that increases HIV acquisition (Hearps et al., 2017). Lack of the vaginal microbiome by antibiotic depletion leads to IL-33 increased production which suppresses IFNγ secretion, leading to Herpes Simplex Virus type 2 (HSV-2) susceptibility due to an impaired antiviral defense (Oh et al., 2016).

**Figure 1:**
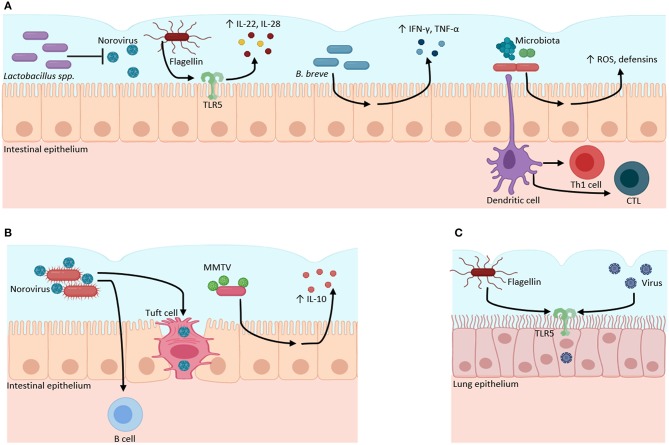
Microbiota–virome interactions in mucosal surfaces. The microbiota has a dual role when it interacts with viruses. **(A)** Microbiota can have a protective role against viral infections. Bacteria from the Lactobacillus genus inhibit Norovirus infection. Bacterial flagellin activates TLR5 to produce inflammatory cytokines (IL-22, IL-18). *B. breve* stimulates the production of pro-inflammatory cytokines (IFN-γ, IL-4, TNFα) against viruses. The gut microbiota regulates the production of ROS and defensins, and the activation of Th1 and CTL against viral infections. **(B)** Microbiota can function as an evasion mechanism, where viruses can bind to bacterial structural molecules (such as LPS) or bacterial pili or membranes to induce immunotolerance through the increase of anti-inflammatory cytokines (IL-10) and to infect host cells. **(C)** Bacterial flagellin increases the infectivity of influenza, Measles, Ebola, Lassa, and Vesicular stomatitis viruses through TLR5 activation in lung epithelial cell culture. Created with BioRender.com.

These findings demonstrate that commensal bacteria in different mucosal sites are part of the antiviral response against pathogenic viruses; nevertheless, there is much yet to define in the mechanisms through which they can achieve this (Figure 1A).

## Microbiota as Promoters of Viral Infections

Despite the significant evidence available about the role of the microbiota in the regulation of the mucosal immune system and the host protection from viral infections, it is also known that, through microbiota rich mucosal surfaces, different viruses enter host cells most efficiently. Furthermore, viruses escape the immune response to establish chronic infections. Then, contrary to the known benefits of gut microbiota, intestinal viruses take advantage of gut bacteria to trigger replication at favorable transmission sites (Kuss et al., 2011).

Human and murine norovirus (MNV) require the presence of bacteria to infect B cells since the lack of both bacteria by antibiotic treatment and B cells in *Rag*^−/−^ mice inhibit the infection by norovirus (Jones et al., 2014; Baldridge et al., 2015). MNV also targets intestinal tuft cells by the CD3001f receptor and antibiotics reduce the specific genes for these cells in the colon. The MNV needs the colonic commensal microbiota to regulate these epithelial cells to utilize them as a reservoir for its chronic infection (Wilen et al., 2018). Commensal bacteria from the human gut, such as *Enterobacter faecium, Klebsiella* spp., *Bacillus* spp., *Bacteroides thetaiotaomicron, L. plantarum*, and *L. gasseri*, among others, bind human norovirus through bacterial pili and membranes, possibly through HBGA-like (histo-blood group antigens) molecules, sialylated gangliosides, and lipopolysaccharides (LPS), which can facilitate the entry of these viruses and the development of the infection (Almand et al., 2017). It is yet to be elucidated the exact mechanisms and molecules this virus utilizes to bind on bacterial surfaces; however, these interactions are a good example of viruses exploiting commensal bacterial to promote their infectivity (Figure 1B).

The intestinal microbiota enhances mouse mammary tumor virus (MMTV), poliovirus, and mammalian orthoreovirus (reovirus) infections (Kane et al., 2011; Kuss et al., 2011). MMTV vertical transmission to offspring's via milk is thought to rely upon TLR4 activation, a PRR for bacteria LPS (Rassa et al., 2002; Jude et al., 2003). The retrovirus MMTV relies on the microbiota interaction to evade the immune response. It binds LPS and induces immune tolerance through a TLR4/MyD88 pathway to induce IL-10 (Kane et al., 2011).

Poliovirus interacts with surface polysaccharides on specific microbes, enhancing host cell binding via the poliovirus receptor (PVR) (Kuss et al., 2011; Robinson et al., 2014). Poliovirus particles are also able to bind to LPS and peptidoglycan (Robinson and Pfeiffer, 2014). Moreover, microbiota-harboring mice support more efficient poliovirus replication (Kuss et al., 2011). Also, the reovirus utilizes bacterial envelope components to enhance virion stability (Berger et al., 2017). Both peptidoglycan and LPS improve viral, and ISVPs (infectious subvirion particles) thermostability; ISVPs are produced when the virus encounters intestinal proteases and play a part in the initial infection steps (Bodkin et al., 1989; Berger and Mainou, 2018).

A recent study showed how human milk oligosaccharides (HMOs) correlate with neonatal RV G10P[11] infection and an increase in abundance of *Enterobacter/Klebsiella*. This neonatal RV evolved to bind HMOs to possibly enter into epithelial cells or to be stabilized by them (Ramani et al., 2018). Considering that HMOs are considered to have a beneficial effect due to their prebiotics effect for bacteria like *Bifidobacterium*, it is remarkable that pathogenic viruses and bacteria can take advantage of this prebiotic to increase their infectivity. Additionally, the commensal microbiota promotes RV infection and affects the immune response to the infection. Antibiotic treatment reduces its infectivity and increases IgA-producing cells, suppressing RV entry (Uchiyama et al., 2014). Vancomycin treatment in healthy adults improves RV vaccine immunogenicity and RV shedding through the increase of Proteobacteria and a reduction in Bacteroidetes (Harris et al., 2018). These studies show that the complete commensal microbiota downregulates the antiviral response to RV infection and only particular taxa can enhance the immunity against viruses.

Recently, it was reported that bacterial flagellin promotes viral infection in an *in vitro* model using lentiviral pseudoviruses encoding the glycoproteins of influenza, Measles, Ebola, Lassa, and Vesicular stomatitis virus in pulmonary epithelial cell culture through TLR5 and NF-κB activation (Benedikz et al., 2019). This finding is particularly exciting since previously, it was reported that flagellin had a protective effect against RV infection in mice (Zhang et al., 2014). The dual effect of flagellin could be due to the differences in the microenvironment and models used to study the interaction between the viruses and bacteria (Figure 1C). These studies exemplify how much is unknown in the interplay of bacteria and viruses.

## Viruses as Part of the Human Microbiome

The intestine contains other types of organisms, besides bacteria, that can influence mucosal and systemic immune responses such as viruses (Minot et al., 2012; Kernbauer et al., 2014; Norman et al., 2015). To interpret the role of the microbiota within viral infections, we must also consider the impact that the virome may play in this interaction. A recent study approximated that in healthy humans, there are 45% of mammalian viruses that are part of the virome without a clinical outcome (Rascovan et al., 2016; Olival et al., 2017). However, similar to bacteria, resident viruses modulate the immune responses (Freer et al., 2018).

Enteric human virome has also been linked to diseases. For example, enteric eukaryotic viruses can be associated with gastroenteritis, enteritis, or colitis (Norman et al., 2015). Bacteriophages perturb the bacterial community, interplay with the host immune system, and an antagonistic relationship between bacteria and bacteriophages during inflammatory bowel disease has been reported (Duerkop and Hooper, 2013; Virgin, 2015). Also, bacteriophages contribute to the spread of antibiotic resistance genes among bacteria; they form a reservoir of these genes within the microbiome (Muniesa et al., 2013; Quirós et al., 2014). In Crohn's disease, a reduction in viral diversity is part of its characteristic dysbiosis (Abeles and Pride, 2014).

Changes in the intestinal virome are significant in AIDS and HIV enteric disease pathogenesis. Reciprocal transactivation between HIV-1 and other human viruses have been reported (White et al., 2006). Monaco et al. found a relation between the enteric adenovirus sequence expansion and the advanced HIV/AIDS stage (Monaco et al., 2016). Also, AIDS alters the commensal plasma virome since an increase in the proportion of anelloviruses has been reported (Li et al., 2013). In this study, the presence of viral sequences from HIV, HCV, hepatitis B virus (HBV), human endogenous retroviruses (HERV), and GB virus C (GBV-C) in the plasma virome of HIV subjects was also found.

HSV-2 may alter vaginal epithelial integrity, which favors HIV infection and transmission (Shannon et al., 2017). Furthermore, it induces genital inflammation and, in the genital tract mucosa, it increases HIV susceptive target cells (Rebbapragada et al., 2007). Epidemiological studies report a coincidence in different populations of women who have a high incidence of HSV-2 infection and an increased HIV risk (Shannon et al., 2017). Viral-bacterial interactions involving HSV, human cytomegalovirus (HCMV), and Epstein–Barr virus type 1 (EBV-1) might contribute to the development of periodontitis, since HSV infects T-lymphocytes and monocytes/macrophages, EBV-1 infects B-lymphocytes, and HCMV infects monocytes/macrophages and T-lymphocytes, which may cause an impaired immune response against bacteria (Contreras and Slots, 2003; Elamin et al., 2017). HSV may promote subgingival attachment and colonization by periodontopathic bacteria using the capsid proteins as receptors for bacteria (Bakaletz, 1995; Contreras and Slots, 2003). This is similar to one of the mechanisms by which commensal bacteria collaborate with viral infections. Chronic periodontitis has also been related to the natural history of HPV in patients with base of tongue cancers (Tezal et al., 2009), since it facilitates the life cycle of HPV infection in the periodontal pocket (Shipilova et al., 2017). This represents a clear example of a virus-bacteria-virus interaction that ends in increased susceptibility to the disease, in this case, head and neck cancer.

## Limitations of Studies Evaluating Bacteria-Viruses' Interactions

It was supposed that bacteria removal, by antibiotics or the lack of these microorganisms in germ-free models, would increase the predisposition to viral infections; on the other hand, it was found that microbiota ablation decreases the infectivity of pathogenic viruses. Experimental systems to evaluate the role of the gut microbiota during enteric viral diseases included two strategies: the infection of germ-free mice and the administration of treatments to eliminate the commensal microbiota in mice prior to a viral infection (Karst, 2016). However, there are several problems with germ-free animals, such as defects in mucosal immune development and changes in intestinal morphology, while the antibiotic treatment has some disadvantages—antibiotics do not remove the entirety of the commensal microorganisms (Wilks and Golovkina, 2012), some gut species are unculturable so its complete absence can't be proved (Schmeisser et al., 2007), there is currently evidence of antimicrobial resistance of some bacterial groups (Pogue et al., 2015).

The study of the effects of the intestinal microbiota on the host immune system requires precisely defined experimental approaches that are complex, and the requirement of samples limits *in vivo* analysis. Also, the study of the microbiome suggests that there is significant variability among individuals, this indicates that microbiomes are dynamic “fingerprints”, though they can change depending on environmental challenges (Bogdanos et al., 2015).

Improvement of *in vitro* and *ex vivo* cultures to simulate more accurately the *in vivo* conditions of microbiome-virome interactions is needed to be able to understand the complexity of this relationship. Otherwise, these models are too simplistic in their approaches, and they should only be used as a first encounter in order to further elucidate the mechanisms of these relations. “Omics” approaches are essential methods to unravel these interactions since pathogenic viruses not only interact with one type of bacteria, but with hundreds of them. Further studies of the relationship between these microorganisms need to take into consideration these approaches to improve our understanding of the complexity of mucosal surface microenvironments.

## Conclusions and Perspectives

Although the role of the host-microbiome in human health has been a topic of interest in recent years, its role in the immune response in the context of the susceptibility to different strains of viruses is an important new consideration. Most viruses access the human body through mucosal surfaces that are traditionally described as rich in a diversity of commensal pathogens. In those sites, viruses interact with hundreds of different commensal bacteria, which are part of the host immune defense. Since the discovery of the protective role of the microbiota, it is easy to imagine that bacteria interact with viruses to eliminate or reduce their infectivity, ensuring the homeostasis of the mucosal sites. However, viruses have developed mechanisms to take advantage of the microbiota, and thereby, evade the immune system. So previous considerations of viruses as the sole grounds of different pathologies is not entirely accurate. It is important to remember the complex interaction within the microenvironment and how they determine the outcomes of disease. Therefore, the commensal microbiota could have a fundamental role against viral infections, but also viruses have evolved to interact with the microbiota, use it, and facilitate viral infection, so based on these observations the microbiota can be in itself a mechanism of viral evasion.

There is also a great need for the development of techniques that allow the characterization of these interactions. There are few translational studies, and the experimental models used have several deficiencies.

In this mini-review, we show how current investigations are just starting to untangle the complex world of the microbiome-virome interactions. While it is undeniable that bacteria aid in the antiviral response to certain viruses, they are also, without a doubt, used as a way of entry by them. This makes it complicated to define the role of the microbiota as a friend or foe in this context.

## Author Contributions

All authors listed have made a substantial, direct and intellectual contribution to the work, and approved it for publication.

### Conflict of Interest Statement

The authors declare that the research was conducted in the absence of any commercial or financial relationships that could be construed as a potential conflict of interest.
